# Notices

**Published:** 1867-10

**Authors:** 


### Notices.

WE commend to our readers as an excellent family paper, edited with care and ability, "THE CONGREGATIONALIST," which has of late absorbed that sterling and time honored name, THE BOSTON RECORDER, published at 15 Cornhill, Boston, Mass., at $3 a year.

"THE ADVANCE," Chicago, Ill., $2 50 a year, has made its appearance. Its mechanical getting up is creditable to all concerned, while the reading matter is various, instructive and timely, and there is no doubt that under the editorial control of such a man as Dr. Patton, it will make its mark for good on our generation.

"THE PROTESTANT CHURCHMAN" is published at $4 per year, at 633 Broadway, New-York; 239 Dock street, Philadelphia, and 35 North Cherry street, Baltimore, with the beautiful motto "Looking to Jesus," maintaining "the lawfulness, the consistency, and the duty of drawing into closer alliance with all evangelical christians, as a safeguard to imperrilled truth." With such an aim, good men of all classes will wish it an abundant success.

"THE UNITED STATES MUSICAL REVIEW" is published at 200 Broadway, by J. L. Peters, promising that each number will give eight pages of choice new music, being six times its price in new music alone. The last number contains: "Kate McFerran," "Maiden's Blush," "Schottish," "Opera Waltz," and "Good Bye, but come Again." by J. R. Thomas.

"THE WITNESS" is "A Paper devoted to the Dissemination of Evangelical Truth," published weekly at $2 a year, by J. Inglis & Co., 26 Cooper Institute, New-York, with the stirring watchword "The night is far spent, the day is at hand."

A. WILLIAMS & Co., Wholesale Dealers in Newspapers, Magazines, Miscellaneous Books, and Stationers, 100 Washington street, Boston, have published "HINTS TO YOUNG MEN," by John Ware, M. D., 65 pages, sent post-paid for forty cents. It was prepared at the request of a committee and published under their direction. It is intended to counteract the mischivous teachings of a certain class of publications on marriage, physiology, &c., and to show our youth a better way and give a more elevated view of the true relation of the sexes.

### *Notices.*

Blackburn Theological Seminary at Carlinville, Illinois, 35 miles north of Alton, on the St. Louis, Alton and Chicago Rail Road, commences the Fall term of the Literary and Theological Departments, September 9th, 1867. We give this notice in affectionate and respectful memory of our old College President, its founder, one of the greatest orators and best men of his time, and so stern an old Roman and Puritan was he, that even General Jackson, his friend and neighbor, was in awe of him.

Developing The Lungs.—Dr. Howe of 227 Grand st., New-York, has sent us an inhaling tube, designed to develope the lungs of those who are "weak chested." It is admirably adapted for its object, as will be explained by the Doctor himself who is a conscientious and good man.

Warming Rooms. — The perfection of a fire for heating a room is the old fashioned, broad, open fire-place filled with hickory wood blazing brightly beautiful of a frosty morning; but there is something better still in the back parlor of No. 2 West 43d st., New-York, which is for the inspection of our subscribers any forenoon. It is the old fashioned fire-place in all respects, except that the back is concave, throwing a great deal more heat out into the room and close on the floor, keeping the feet warm. In this contrivance, wood, peat, coke or coal, hard or soft, can be burned at pleasure. To keep up a wood fire on a cold day requires the services of one person nearly all the time; in this plan of room-warming the fire is built in the morning, and requires no further attention until noon, giving all the time a broad bed of burning coals, aglow with life and comfort. It kindles the hardest coal without a blower and with proper management, gives very little dust in a whole day's burning. The ashes fall through a grating into the cellar, leaving all the cinders behind to be fully consumed, hence there is no waste. This is Dixon's Low Down Grate and there is nothing equal to it anywhere. It can be put in the place of a common grate with very little trouble and dirt in a few hours. Call on Mead and Woodward, 37 Park Row, New-York, or Dixon and Sons, 1324 Chestnut st., Philadelphia, Pa.

